# Unveiling the Stable Semiconducting 1T′-HfCl_2_ Monolayer: A New 2D Material

**DOI:** 10.1021/acsomega.4c10560

**Published:** 2025-03-28

**Authors:** Celso Alves Do Nascimento Júnior, Elie Albert Moujaes, Maurício Jeomar Piotrowski, Celso Ricardo Caldeira Rêgo, Diego Guedes-Sobrinho, Luiz Antônio Ribeiro Júnior, Teldo Anderson da Silva Pereira, Alexandre Cavalheiro Dias

**Affiliations:** † Institute of Physics, University of Brasília, Brasília, Federal District 70919-970, Brazil; ‡ Physics Department, Federal University of Rondônia, Porto Velho 76801-974, Brazil; § Institute of Physics, Solid State Physics Department, Federal University of Bahia, Salvador, Bahia 40170-115, Brazil; ∥ Department of Physics, Federal University of Pelotas, PO Box 354, Pelotas, Rio Grande do Sul 96010-900, Brazil; ⊥ Karlsruhe Institute of Technology (KIT), Institute of Nanotechnology, Hermann-von-Helmholtz-Platz, Eggenstein-Leopoldshafen 76344, Germany; # Chemistry Department, Federal University of Paraná, Curitiba CEP 81531-980, Brazil; ∇ Computational Materials Laboratory, LCCMat, Institute of Physics, University of Brasília, Brasília 70910-900, Brazil; ○ Physics Graduate Program, Institute of Physics, Federal University of Mato Grosso, Cuiabá, Mato Grosso 78060-900, Brazil; ◆ National Institute of Science and Technology on Materials Informatics, Campinas 13083-100, Brazil; ¶ Institute of Physics and International Center of Physics, University of Brasília, Brasília 70919-970, Federal District Brazil

## Abstract

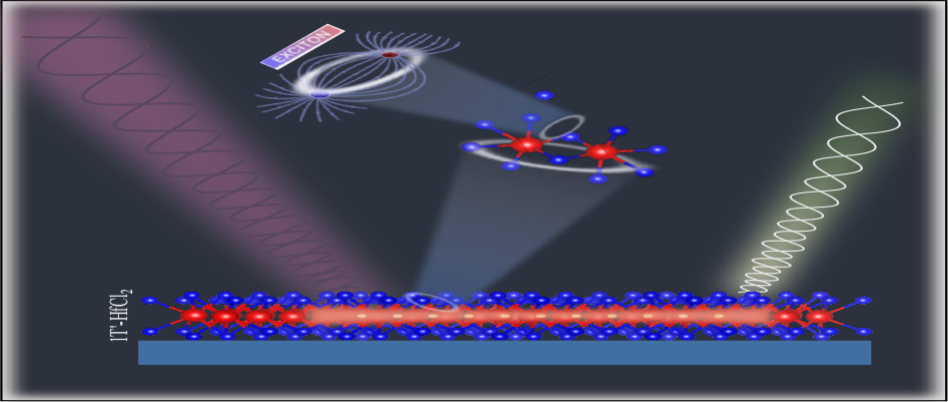

Designing novel 2D materials is crucial for advancing next-generation optoelectronic technologies. This work introduces and analyzes the 1T′-HfCl_2_ monolayer, a novel low-symmetry variant within the 2D transition metal dichloride family. Phonon dispersion calculations reveal no imaginary frequencies, suggesting its dynamical stability. 1T′-HfCl_2_ exhibits semiconducting behavior with a direct band gap of 1.52 eV, promising for optoelectronics. Strong excitonic effects with a binding energy of 525 meV highlight significant electron–hole interactions typical of 2D systems. Furthermore, the monolayer achieves total reflection of linearly polarized light along the *ŷ* direction at photon energies above 2.5 eV, showcasing its potential as an optical polarizing filter. Raman spectra calculations also reveal distinct peaks between 96.72 and 270.38 cm^–1^. The tunable excitonic and optical properties of 1T′-HfCl_2_ highlight its potential in future functional devices, paving the way for its integration into semiconducting and optoelectronic applications.

## 1 Introduction

With the rise of graphene in the early 21st century,^1,2^ a new frontier in materials research emerged: the study of two-dimensional (2D) materials and their novel properties arising from quantum confinement in the nonperiodic direction,^3^ which has unveiled a broad spectrum of their remarkable properties and functionalities.^4−8^ Due to their atomic thickness, 2D materials can be patterned horizontally using chemical and mechanical techniques,^9^ allowing their monolayers to be combined into van der Waals (vdW) heterojunctions for property tuning.^10−12^

2D materials properties, including high surface area, mechanical flexibility, and tunable electronic and optical properties, have garnered significant interest.^13,14^ Researchers are actively exploring new 2D materials to manipulate their characteristics for applications in sensing and catalysis.^15^ The ability to design and stack monolayers provides huge possibilities for creating devices with customizable properties.^16^ These properties can be fine-tuned to meet the needs of emerging technologies, offering solutions to challenges across various fields.^17^

Among 2D materials, transition metal dichalcogenides (TMDs) have gained considerable attention for two reasons,^7,8,18^ due to their similarity with graphene, adopting the honeycomb structure known as the 2H phase;^7^ and semiconducting behavior.^1,2^ TMDs consist of a transition metal (M) sandwiched between two chalcogens (X) with the formula MX_2_, forming layers with hexagonal symmetry. The widely studied 2H phase,^7^ TMDs can adopt 1T^19^ and the 1T′ phases.^20,21^ For TMDs based on Mo and W, the 1T phase is unstable in its free-standing form;^19,22^ however, it can be stabilized through a spontaneous Peierls distortion along the *x̂* direction, forming a 2 × 1 × 1 distorted supercell known as 1T′ phase.^22^ This phase features one-dimensional (1D) zigzag chains along the *ŷ* direction.^22^

The 1T′ phase has attracted attention due to its topological properties and potential applications in electronic and spintronic devices.^20,21^ The transition phases between 1T and 1T′ can be induced through methods such as chemical doping and pressure,^23−25^ providing a dynamic platform for exploring new physical phenomena and pioneering advanced technologies. For example, 1T′ Mo and W based TMD monolayers have been proposed as candidates for the quantum spin Hall (QSH) effect due to the overlap of metal-*d* conduction bands and chalcogenide-*p* valence bands.^20,26^ This band localization across different layers allows topological electronic properties to be controlled using an external electric field, which is highly desirable for vdW devices.^20^ While Varsano and coworkers observed excitonic insulator behavior in the 1T′ MoS_2_ monolayer,^26^ Barbosa and coworkers found traditional semiconductor behavior in the 1T′ WSe_2_ monolayer.^27^

Based on these findings, attention has shifted to transition metal dihalides (TMDHs), driven by the discovery of materials with properties similar to TMDs. TMDHs follow the chemical formula MY_2_, where M is a transition metal and Y represents halides such as Cl, Br, or I.^28,29^ Previous studies have shown that single-layer PbI_2_ is stable and exhibits remarkable excitonic and spin–orbit coupling effects.^30,31^ More recently, interest has extended to 2D IVB-VIIA group transition metal halides due to their novel electronic and topological properties, including the quantum spin Hall effect and large nontrivial band gaps.^32^ These features make them promising candidates for advanced electronic, spintronic, and optoelectronic applications. Apart from the 1T′ phase, and despite the significant gap in the literature, Huang and coworkers made important advancements in the field by proposing a series of 2D HfX_2_ (X = Cl, Br, I) monolayers and related type-II van der Waals (vdW) heterostructures. Using first-principles calculations, they demonstrated that HfY_2_ monolayers are both dynamically and thermodynamically stable, with band gaps ranging from 0.9 to 1.7 eV, an optimal range for donor systems in excitonic solar cells (XSCs).^33^ These materials also exhibit high visible light absorption (10^5^ cm^–1^) and notable power conversion efficiencies (PCEs) of 17.150%, 21.438%, and 20.439% in type-II vdW heterostructures, highlighting their potential for solar energy conversion.^33^ While the 1T′ phase is considered stable, further systematic investigations are necessary to identify other possible phases and evaluate their energetic stability.

Despite these advancements, little attention has been given to the 1T′ phase of TMDHs involving group IV transition metals, such as Ti, Zr, and Hf. This gap presents an opportunity to explore the 1T′ HfCl_2_ monolayer, focusing on its electronic, vibrational, optical, and excitonic properties. By using density functional theory (DFT), we characterize the material’s structural and electronic properties. We aim to deepen our understanding of this low-dimensional material, uncovering new opportunities for its application in emerging technologies. We first perform phonon dispersion calculations to assess the dynamical stability of the 1T′-HfCl_2_ monolayer. The Raman and infrared (IR) spectra provide further insights into its structural and vibrational properties. We then investigate its electronic properties using the Perdew–Burke–Ernzerhof (PBE) and Heyd–Scuseria–Ernzerhof (HSE06) exchange-correlation functionals, considering spin–orbit coupling (SOC) effects. Finally, we evaluate the optical properties, such as the absorption coefficient, refractive index, and reflectivity, within the Independent Particle Approximation (IPA) and account for excitonic effects using the Bethe–Salpeter Equation (BSE).^34^

## 2 Methodology and Computational Details

First-principles calculations were performed using the Vienna Ab Initio Simulation Package (VASP) within the framework of DFT,^35,36^ to investigate the structural, electronic, vibrational, and optical properties of the 1T′ HfCl_2_ monolayer.^37,38^ To analyze the electronic and structural properties, we employed the Perdew–Burke–Ernzerhof (PBE) functional,^39^ a semilocal exchange-correlation functional based on the Generalized Gradient Approximation (GGA).^40^ PBE has been shown to offer a good balance between computational efficiency and accuracy in predicting crystalline structures when compared with experimental data.^41^ However, it is well-documented that semilocal functionals tend to underestimate the band gap due to self-interaction errors.^42−46^ To mitigate this issue, we employed the Heyd–Scuseria–Ernzerhof (HSE06) hybrid functional,^47,48^ which provides a more accurate prediction of the fundamental band gap.

The Kohn–Sham (KS) equations were solved using the projector augmented-wave (PAW) method.^49,50^ Structural optimizations were carried out by minimizing the interatomic forces, with a plane-wave cutoff energy of 463.55 eV. The convergence criterion for the atomic forces was set to less than 0.010 eV Å^–1^, and the self-consistent field (SCF) calculations used an energy convergence threshold of 10^–6^ eV. For Brillouin zone (BZ) integrations, a **k**-mesh of 6 × 14 × 1 was employed for all electronic and vibrational properties, except for the density of states (DOS), for which a 12 × 28 × 1 **k**-mesh was used. Phonon and thermodynamic properties were computed using the Phonopy package^51^ in conjunction with VASP. Phonon dispersion was determined using density functional perturbation theory (DFPT),^52^ with a 2 × 2 × 1 supercell and a 3 × 7 × 1 **q** (phonon)-mesh. A vacuum thickness of 18.63 Å was added along the *z*-axis to avoid interactions with adjacent monolayer images.

The excitonic and optical properties within the independent particle approximation (IPA) and Bethe–Salpeter equation (BSE),^34^ were computed using the WanTiBEXOS code.^53^ Single-particle electronic levels were determined through a maximally localized Wannier functions scheme obtained from the HSE06 calculations using the Wannier90 (W90) package,^54^ focusing on the Hf d-orbitals and Cl p-orbitals. The BSE was solved with a 2D Truncated Coulomb Potential (V2DT),^55^ considering the 9 lowest conduction bands and the 6 highest valence bands, utilizing a **k**-mesh of 19 × 41 × 1 and a smearing value of 0.01 eV to ensure result precision.

Raman spectra were calculated using the QERaman code^56^ interfaced with Quantum Espresso (QE).^57−59^ An electronic grid of 8 × 16 × 1 was used to achieve converged electron-photon and electron–phonon matrix elements necessary for determining the Raman intensities.

## 3 Results and Discussion

### 3.1 Structural Stability and Thermodynamical Properties

**Figure 1:**
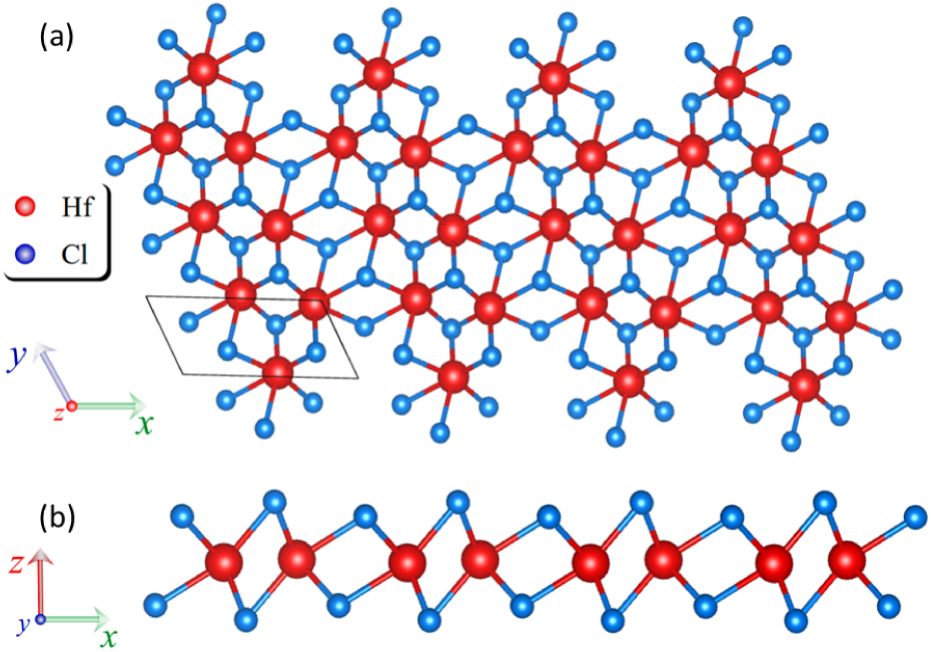
(a) Top and (b) side views of the 1T′-HfCl_2_ monolayer crystal structure. The Hf atoms are depicted as red spheres, while Cl atoms are represented by blue spheres.

The HfCl_2_ monolayer under investigation features a distorted 1T′ crystal structure comprising 2 Hf and 4 Cl atoms. This structure belongs to the triclinic *P*_1_ space group and exhibits a *C*_1_ point group symmetry. The 1T′-crystalline phase is characterized by a Peierls-distorted octahedral coordination, akin to the 1T-crystalline phase, resulting in varying Hf–Cl bond lengths within the same unit cell. Figure 1 illustrates the crystalline structure, with (a) showing the top view and (b) the side view of the 1T′-phase of the HfCl_2_ monolayer. The unit cell is defined by a triclinic Bravais lattice, with lattice parameters *a*_0_ = 6.97 Å and *b*_0_ = 3.27 Å in the (*xy*)-plane, forming an intervector angle of 118°. The lattice parameter along the *z*-axis is *c*_0_ = 18.63 Å, which ensures sufficient vacuum in the nonperiodic direction of the 2D material to minimize interactions between adjacent periodic images.

**Figure 2:**
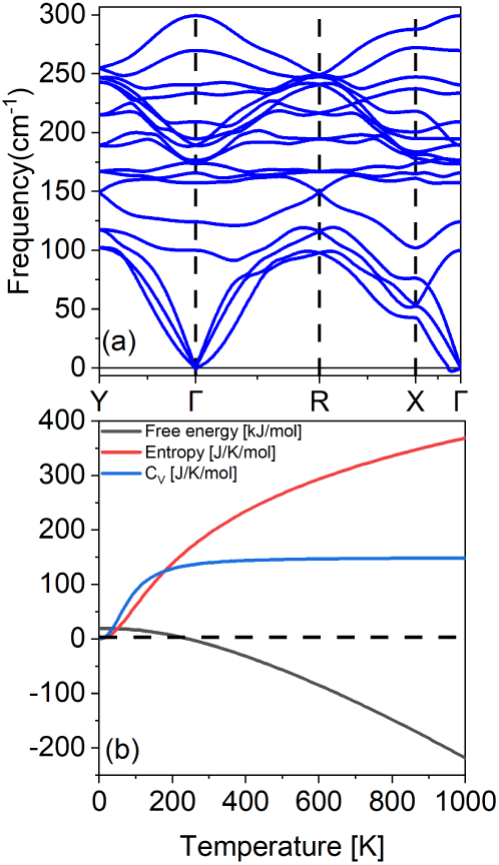
(a) Phonon dispersion and (b) thermodynamic properties: Gibbs free energy, entropy, and heat capacity at constant volume of the 1T′-HfCl_2_ monolayer.

The phonon dispersion curve, depicted in Figure 2a, confirms the dynamical stability of the HfCl_2_ monolayer. The absence of imaginary frequencies across all high-symmetry paths (Y−Γ–R–X−Γ) indicates that the material is free from vibrational instabilities, thus suggesting its capability to maintain structural integrity under finite-temperature conditions.

Thermodynamic properties are illustrated in Figure 2b. The entropy (red line) shows an approximately linear increase with temperature up to 300 K, followed by a slower, nonlinear growth. This behavior reflects the increasing disorder and the contribution of high-energy phonon modes at elevated temperatures. The heat capacity (*C*_V_, blue line) exhibits the expected *T*^3^ dependence at low temperatures, consistent with predictions from the Debye model. As the temperature rises, the heat capacity approaches the Dulong–Petit limit of around 400 K, indicating that all vibrational modes are thoroughly excited.

The Gibbs free energy (black line) decreases steadily with increasing temperature, becoming negative around 300 K. This observation implies that the HfCl_2_ monolayer is thermodynamically favorable at room temperature, positioning it as a promising candidate for experimental synthesis. The negative free energy at ambient temperatures further supports the material’s anticipated stability under standard environmental conditions.

**Figure 3:**
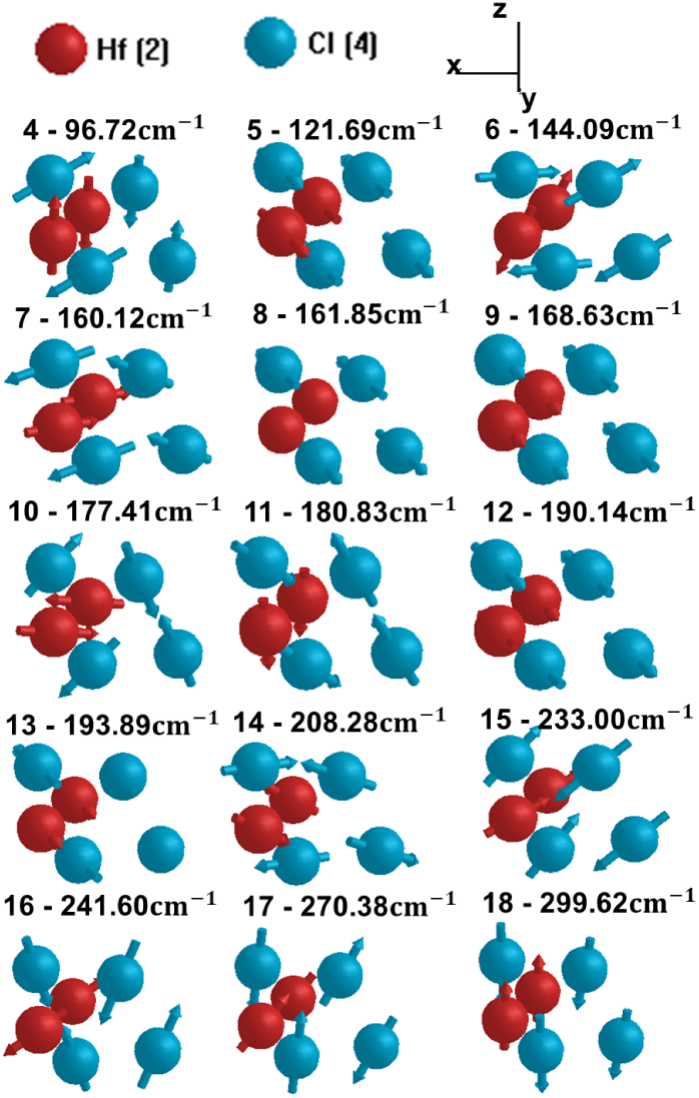
Optical vibrational modes of the 1T′-HfCl_2_ monolayer at the Γ point. The Hf (Cl) atomic species are represented by red (blue) spheres.

The system exhibits 18 vibrational modes: 3 acoustic modes, characterized by coherent atomic vibrations in the same direction, and 15 optical modes, representing more complex vibrational patterns involving varying relative motions among the atoms. Figure 3 shows the optical vibration modes of the 1T′-HfCl_2_ monolayer at the Γ point of the first BZ. In these visualizations, Hf atoms are represented by red spheres, while Cl atoms are denoted by blue spheres. Each mode corresponds to specific atomic displacements characterized by distinct motions between the Hf and Cl atoms.

These optical modes highlight potential phonon–phonon interactions, which are critical for predicting thermal transport properties in this 2D material.

Low-frequency optical modes mainly involve coordinated movements between heavier Hf and lighter Cl atoms, which typically result in out-of-phase oscillations. Due to the larger mass of Hf atoms, these modes manifest at lower frequencies. In contrast, high-frequency optical modes are dominated by oscillations of lighter Cl atoms, which exhibit faster vibrations due to their smaller mass. These modes reflect localized vibrational patterns in which Cl atoms move more vigorously than the Hf atoms. The optical modes at the Γ-point are crucial for understanding the material’s infrared and Raman spectra, as they determine how the system interacts with external electromagnetic fields and light scattering. Some of these modes are expected to be IR- or Raman-active, providing valuable information for experimental validation of the monolayer, as will be discussed in the following section.

### 3.2 Vibrational Raman and Infrared Spectra

In principle, the irreducible optical phonon modes of the *P*_1_ space group at the Γ high symmetry point decompose into:
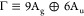
where the gerade (A_g_) symmetries are Raman active and all ungerade (A_u_) modes are infrared active.

**Figure 4:**
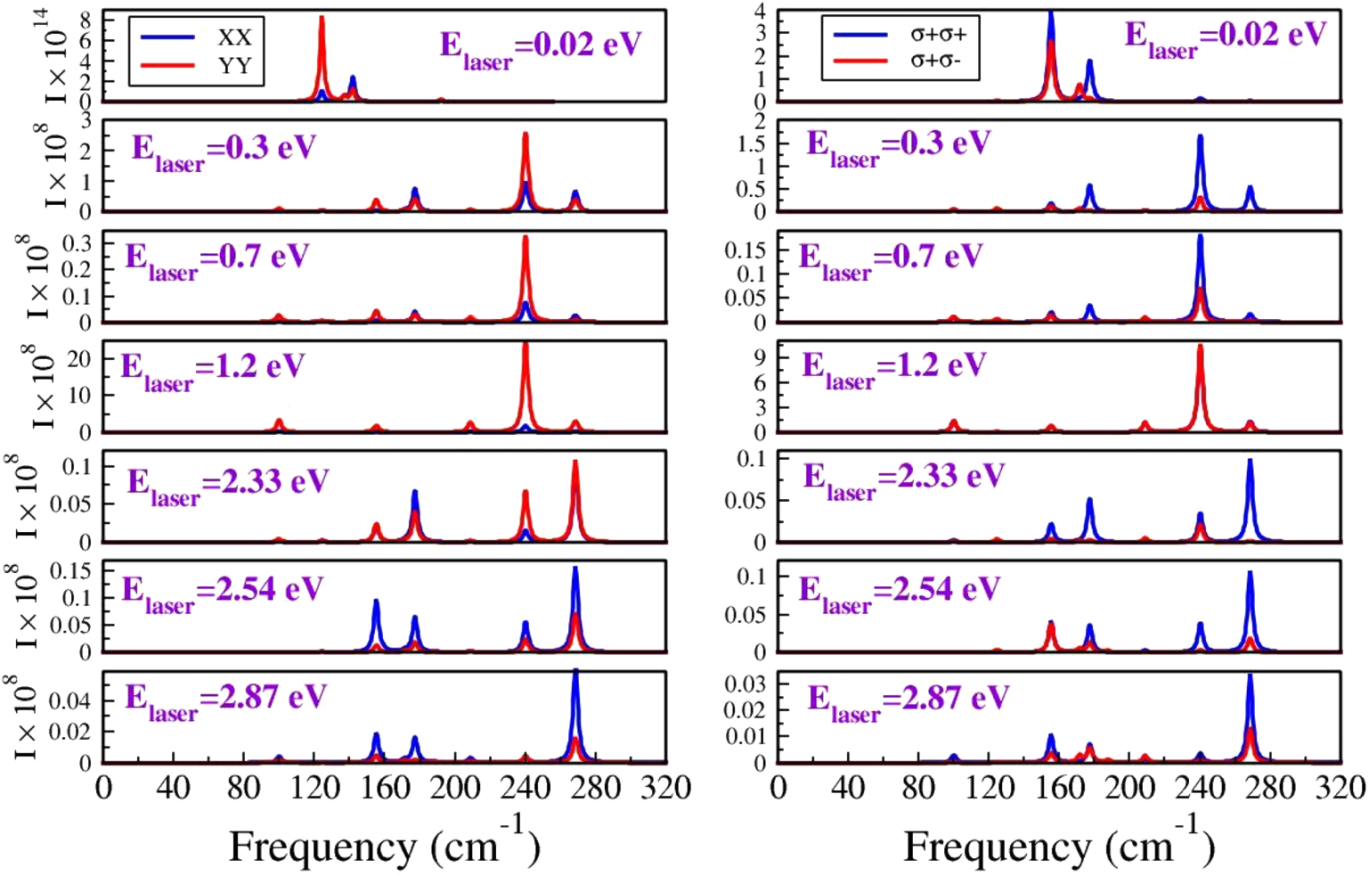
Raman spectra of the 1T′-HfCl_2_ monolayer at various laser energy (*E*_laser_) values for both linearly (*XX* and *YY*) and circularly polarized light.

**Figure 5:**
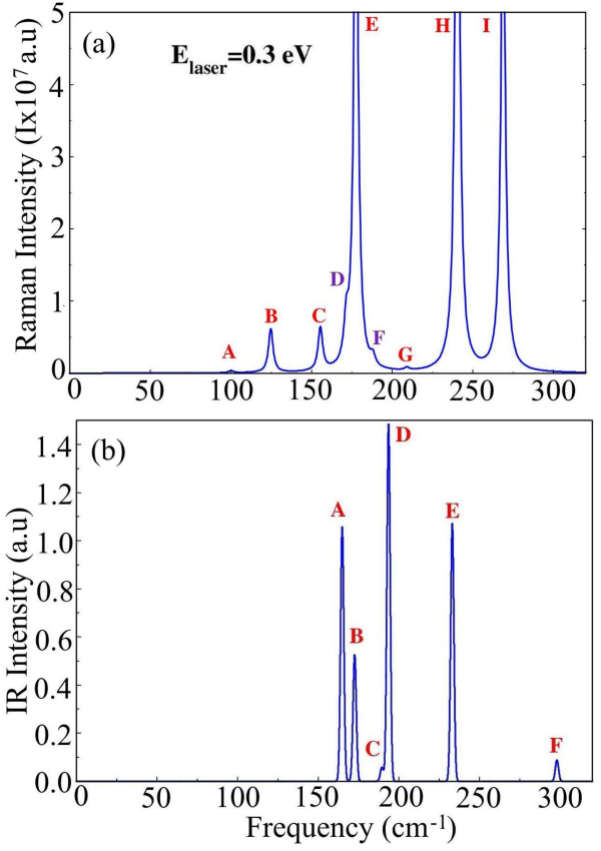
(a) Raman spectra for a 0.3 eV laser energy showing all nine modes including the ″hidden″ 161.85 cm^–1^ and 180.83 cm^–1^ modes, labeled as D and F (in violet). (b) IR spectra of the 1T′-HfCl_2_ monolayer. Peaks A, B, C, D, E, and F correspond to the IR frequencies 160.12 cm^–1^, 168.63 cm^–1^, 190.14 cm^–1^, 193.89 cm^–1^, 233.00 cm^–1^, and 299.63 cm^–1^, respectively.

The evolution of the Raman (R) active modes for several laser energies (*E*_laser_) within the infrared to visible regime is illustrated in Figure 4. The intensity of a peak located at ∼96.72 cm^–1^ increases as we traverse the infrared region but subsequently decreases within the visible range, only to reemerge at *E*_laser_ = 2.87 eV. In general, seven Raman peaks can be identified at the following frequencies: 96.72 cm^–1^, 121.69 cm^–1^, 144.09 cm^–1^, 177.41 cm^–1^, 208.28 cm^–1^, 241.60 cm^–1^, and 270.38 cm^–1^. These correspond to optical modes 4, 5, 6, 10, 14, 16, and 17 depicted in Figure 3. The remaining two modes at 172.36 cm^–1^ and 188.00 cm^–1^ are less intense peaks, merged with the 177.41 cm^–1^ sharp peak. They can be easily detected on a smaller scale graph for a 0.3 eV laser energy (Figure 5a).

Furthermore, our calculations reveal that the following optical modes are infrared active: mode 7 in 160.12 cm^–1^, mode 9 at 168.63 cm^–1^, mode 12 at 190.14 cm^–1^, mode 13 at 193.89 cm^–1^, mode 15 at 233.00 cm^–1^, and mode 18 at 299.63 cm^–1^. The intensities of these IR-active modes are presented in Figure 5b. Table 1 summarizes the above results.

**Table 1:** Frequencies, in cm^–1^, and Symmetries of Raman and IR Vibrational Active Modes in 1T′-HfCl_2_ Monolayer^a^

Frequency (cm^–1^)	Raman	IR
96.72	^1^A_g_	
121.69	^2^A_g_	
144.09	^3^A_g_	
160.12		^1^A_u_
168.63		^2^A_u_
172.35	^4^A_g_	
177.41	^5^A_g_	
188.00	^6^A_g_	
190.14		^3^A_u_
193.89		^4^A_u_
208.28	^7^A_g_	
233.00		^5^A_u_
241.60	^8^A_g_	
270.28	^9^A_g_	
299.63		^6^A_u_

a The symmetry of Raman or IR inactive modes is left blank.

This analysis underscores the distinct vibrational characteristics of the 1T′-HfCl_2_ monolayer, revealing how its optical properties change with varying laser energies. The coexistence of both Raman-active and infrared-active modes emphasizes the material’s versatility for potential applications in optoelectronic devices and advanced sensing technologies, where understanding the vibrational modes is crucial for tailoring its performance.

**Figure 6:**
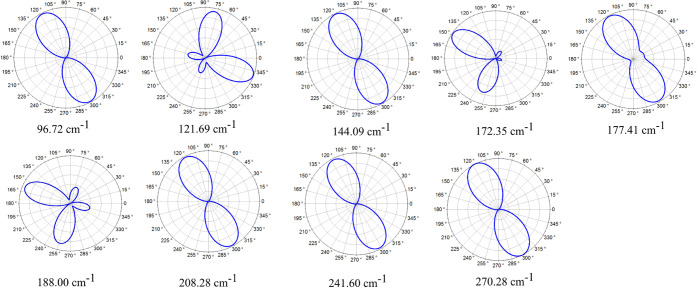
Polar intensity plots of the Raman modes for circularly polarized light at *E*_laser_ = 2.33 eV.

For laser energy of 2.33 eV, Figure 6 presents polar plots illustrating the intensity of the R-active modes, explicitly detailing how the intensity varies as a function of direction within the monolayer. The intensity variation exhibits a similar pattern for modes 4, 6, 10, 14, 16, and 17, with intensity maxima occurring at angles 117.94° and 297.94°. In contrast, mode 5 reaches its maximum intensity at an angle of 339.76°, with the second highest intensity observed at 78.33°. None of the modes display their highest intensity along the *x̂* or the *ŷ* directions, indicating that the vibrational characteristics of these modes are anisotropic and depend significantly on the angle of observation.

This directional dependence of the Raman intensity highlights the complex interplay between the lattice symmetry and the vibrational modes in the 1T′-HfCl_2_ monolayer, suggesting that the material’s optical properties can be precisely tuned by adjusting the orientation of the incident light. Understanding these variations is crucial for potential applications in sensors and photonic devices, where orientation and polarization of light play critical roles in performance.

### 3.3 Electronic Properties

**Figure 7:**
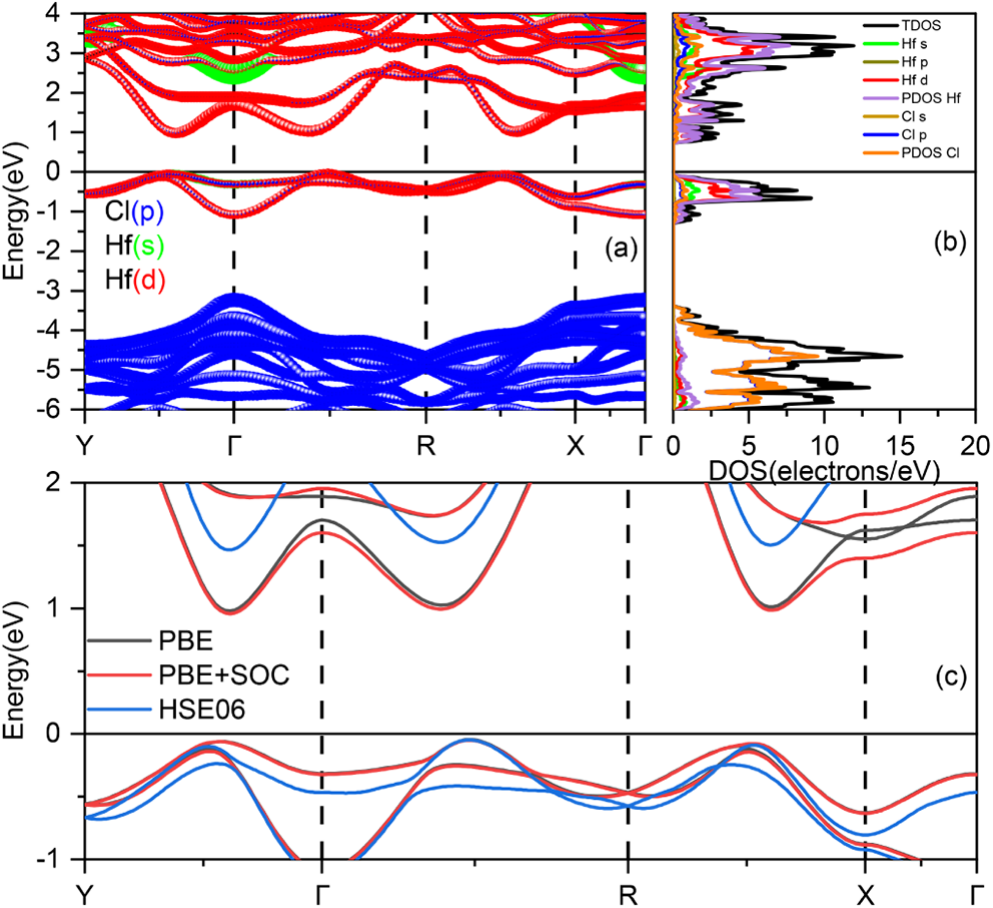
(a) Electronic band structure and (b) projected density of states (DOS) of the 1T′-HfCl_2_ monolayer at the PBE level. (c) PBE calculation of the band structures with and without SOC. (d) Comparison between the PBE and HSE06 band structures of 1T′-HfCl_2_. The Fermi level is set at 0 eV.

From the orbital-projected band structure depicted in Figure 7a, it is evident that the 1T′-HfCl_2_ monolayer exhibits a semiconductor behavior, characterized by a band gap present between the high-symmetry points Γ and *X* (and also between *Y* and Γ). The orbital-projected density of states shown in Figure 7b indicates that, near the Fermi level, the predominant contributions originate from the Hf d-orbitals and Cl p-orbitals. This observation aligns with Figure 7a), where the Hf orbitals are primarily responsible for the electronic states close to the Fermi level.

A comparison of the PBE and PBE + SOC electronic band structures, illustrated in Figure 7c, reveals that including SOC does not significantly influence the electronic band gap. However, SOC does lead to band splitting at the *X* high-symmetry point within the conduction states. We will, therefore, neglect the SOC effects in the subsequent HSE06 calculations. A comparison between the PBE and HSE06 electronic band structures, as shown in Figure 7d, indicates that the HSE06 functional significantly enhances the electronic band gap. The calculations yield a fundamental band gap of 1.45 eV and a direct band gap of 1.52 eV in the HSE06 results, representing an increase of 0.5 eV compared to the fundamental band gap obtained using the PBE functional. This enhancement in the band gap is crucial for applications in electronic and optoelectronic devices, suggesting that the HSE06 functional provides a more accurate description of the electronic properties of the 1T′-HfCl_2_ monolayer.

### 3.4 Optical and Excitonic Properties

**Figure 8:**
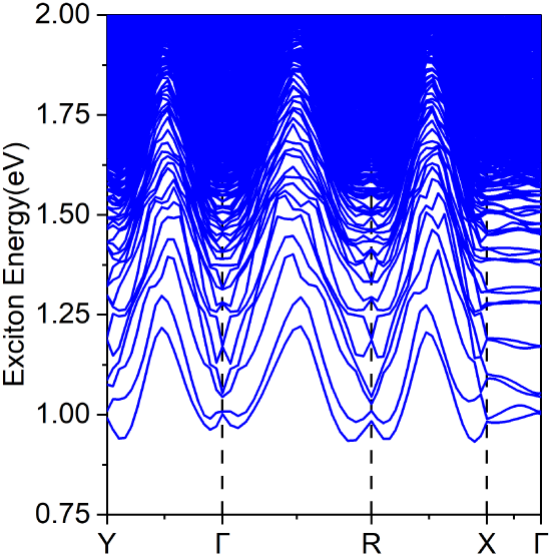
Exciton band structure of the 1T′-HfCl_2_ monolayer, obtained using the MLWF-TB + BSE at the HSE06 parametrization level.

The excitonic band structure depicted in Figure 8 reveals both direct (at Γ) and indirect (at any other **k**-point) excitonic states, derived from the solution of the BSE. In contrast to the electronic band structure, this representation does not allow for a straightforward classification of the exciton bands into conduction and valence states. This is because the excitonic levels arise from the energy differences between conduction and valence states and the Coulomb interaction potential that binds the electron–hole pairs.

Our findings indicate that the exciton ground state is indirect, with an energy value of 0.93 eV, leading to an exciton binding energy of 525 meV. This value differs between the fundamental electronic band gap and the exciton ground state energy. This binding energy is slightly higher than the typical range observed for 2D materials, which is usually between 100 and 500 meV.^7,60^ This exciton binding energy is comparable to or slightly higher than those observed in other 2D materials commonly explored for similar applications. For example, monolayer MoS_2_, a well-studied transition metal dichalcogenide, exhibits exciton binding energies around 450 meV, while hexagonal boron nitride (hBN) demonstrates a quasiFrenkel character, with stronger excitonic effects and binding energies exceeding 700 meV.^61^ The value reported for 1T′-HfCl_2_ places it in a range where excitonic effects significantly influence the optical properties, indicating its potential for optoelectronic applications that benefit from enhanced light-matter interactions.

In addition, the direct excitonic ground state, which corresponds to the optical band gap of the system, is measured at 1.52 eV. This is in contrast with the behavior observed in the 1T′-MoS_2_ material, classified as an excitonic insulator,^26^ where the exciton binding energy exceeds the electronic band gap.^62^ In fact, the 1T′-HfCl_2_ monolayer behaves as a semiconductor, akin to the 1T′-WSe_2_ monolayer.^27^ This distinction is pivotal for elucidating the material’s electronic properties and informing its prospective applications in optoelectronic devices.

**Figure 9:**
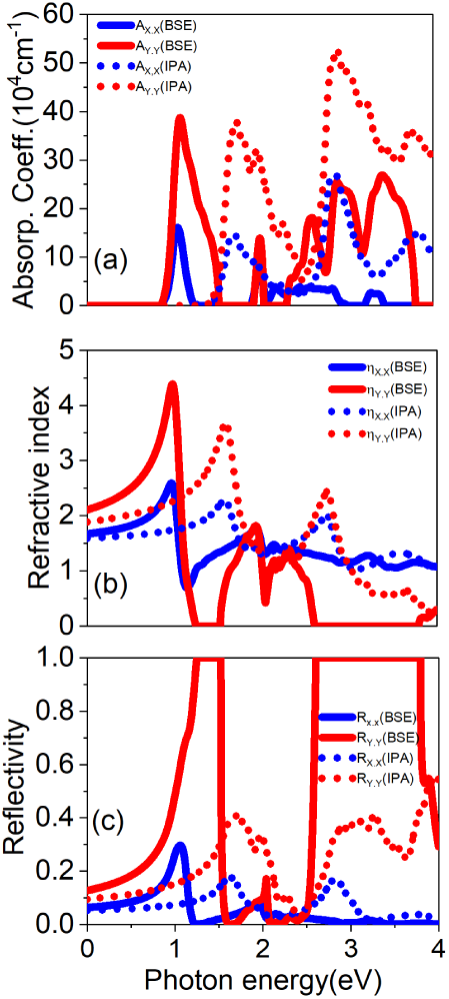
Optical properties of the 1T′-HfCl_2_ monolayer: (a) absorption coefficient, (b) refractive index, and (c) reflectivity, calculated at the BSE (represented by solid blue and red curves) and the IPA levels (represented by blue and red dotted curves). These properties are evaluated for light polarized in the *x̂* (blue curves) and *ŷ* directions (red curves).

Figure 9 depicts the linear optical response of the 1T′-HfCl_2_ monolayer, highlighting the differences between the results obtained with and without excitonic effects. In panel (a), a pronounced optical anisotropy is evident in the absorption spectrum, characterized by higher absorption coefficients along the *ŷ* polarization direction. This behavior is attributed to the significant exciton binding energy of 525 meV, significantly altering the optical band gap. More specifically, the optical band gap is approximately 1.5 eV at the IPA level and around 0.9 eV when incorporating excitonic effects through the BSE.

Figure 9b,c presents the refractive index and reflectivity, respectively. The refractive index reaches a maximum value of 4.5 at the BSE level when light is polarized along the *ŷ* direction at an energy near 0.9 eV. Conversely, at the same energy, the refractive index is measured to be 2.5 for light polarized along the *x̂* direction. A similar trend is observed at the IPA level, albeit with the peak refractive index blue-shifted to 1.5 eV. Optical excitations exceeding 1.0 eV, including excitonic effects, result in a lower refractive index than the IPA predictions.

The optical anisotropy seen in both the absorption coefficient and the refractive index is further corroborated by the reflectivity plot in Figure 9c. At the BSE level, the reflectivity remains at 100% for photon energies in the ranges of 1.15–1.50 eV and 2.60–3.90 eV when polarized along the *ŷ* direction. Subsequently, the reflectivity experiences a sharp drop to 0 beyond this range. This linear optical response indicates that the 1T′-HfCl_2_ monolayer holds promise as a highly effective polarizing filter. Its capability to reflect light in the *ŷ* direction within the ultraviolet range and a small segment of the infrared spectrum positions it as a potential candidate for applications in photonic devices that require selective polarization control.

## 4 Conclusion

This study presents a detailed analysis of the structural, electronic, vibrational, optical, and excitonic properties of the 1T′-HfCl_2_ monolayer. The unit cell exhibits equilibrium lattice constants of *a*_0_ = 6.97 Å and *b*_0_ = 3.27 Å, characteristic of its structural properties. The absence of imaginary frequencies in the phonon dispersion spectrum confirms that the system is stable thermodynamically. Additionally, the Gibbs free energy, which becomes negative near room temperature, indicates the feasibility of experimental synthesis.

The 1T′-HfCl_2_ monolayer behaves as a direct band gap semiconductor with a band gap of 1.52 eV, accurately determined using the hybrid HSE06 functional. The primary contributions to the electronic states around the Fermi level stem from the Hf d-orbitals and the Cl orbitals present only at lower valence states. Raman spectra reveal seven distinct peaks, with the lowest occurring at 96.72 cm^–1^ and the highest at 270.38 cm^–1^. Under reasonable experimental conditions, the intensity of these Raman active modes reaches its maximum at approximately 120° within the monolayer plane.

Regarding excitonic and optical properties, we conclude that the 1T′-HfCl_2_ monolayer does not function as an excitonic insulator, as seen in the 1T′-MoS_2_ system. Instead, it behaves as a conventional semiconductor with significant excitonic effects, including an exciton binding energy of 525 meV and an optical band gap of 0.93 eV when quasi-particle effects are considered. The system also exhibits optical anisotropy for linearly polarized light, particularly for the refractive index, which favors the *ŷ* polarization direction except for photon energies exceeding 3.05 eV, where the *x̂* polarization dominates. This anisotropic behavior extends to the reflectivity graphs, suggesting potential applications of this material as a polarizing filter due to its strong reflectivity along the *ŷ* direction.

The remarkable characteristics of the 1T^′^-HfCl_2_ monolayer underline its immense potential in nanoelectronics and optoelectronics, making it a strong contender for groundbreaking technological advancements. This study opens the door for exploring novel applications for this fascinating 2D material.
